# Permeation Mechanisms in the TMEM16B Calcium-Activated Chloride Channels

**DOI:** 10.1371/journal.pone.0169572

**Published:** 2017-01-03

**Authors:** Simone Pifferi

**Affiliations:** Neurobiology Group, SISSA, Scuola Internazionale Superiore di Studi Avanzati, Trieste, Italy; Universidad Autonoma de San Luis Potosi, MEXICO

## Abstract

TMEM16A and TMEM16B encode for Ca^2+^-activated Cl^−^ channels (CaCC) and are expressed in many cell types and play a relevant role in many physiological processes. Here, I performed a site-directed mutagenesis study to understand the molecular mechanisms of ion permeation of TMEM16B. I mutated two positive charged residues R573 and K540, respectively located at the entrance and inside the putative channel pore and I measured the properties of wild-type and mutant TMEM16B channels expressed in HEK-293 cells using whole-cell and excised inside-out patch clamp experiments. I found evidence that R573 and K540 control the ion permeability of TMEM16B depending both on which side of the membrane the ion substitution occurs and on the level of channel activation. Moreover, these residues contribute to control blockage or activation by permeant anions. Finally, R573 mutation abolishes the anomalous mole fraction effect observed in the presence of a permeable anion and it alters the apparent Ca^2+^-sensitivity of the channel. These findings indicate that residues facing the putative channel pore are responsible both for controlling the ion selectivity and the gating of the channel, providing an initial understanding of molecular mechanism of ion permeation in TMEM16B.

## Introduction

Ca^2+^-activated Cl^−^ channels (CaCCs) are widely expressed in different cell types where they play a variety of important physiological roles. A classical example of the CaCC’s function is that of some amphibian oocytes where they block the polyspermy [1]. In olfactory and vomeronasal sensory neurons, CaCCs mediate a large component of transduction current [2–5] and in other neuronal cell types they can control excitability [6]. Moreover, they regulate the fluid transport in different types of epithelia [7] and modulate the activity of smooth muscles of the blood vessels [8,9].

CaCCs are interesting because of their various hallmark features. In particular, they are directly gated by sub-micromolar/micromolar concentrations of intracellular Ca^2+^ and the apparent Ca^2+^-sensitivity depends on membrane voltage [10]. At low [Ca^2+^]_i_ CaCCs show a voltage-dependent outward rectifying conductance whereas, at higher concentrations, the current becomes leak-like with an ohmic relation. Finally, the pore of CaCCs shows a relatively poor selectivity among anions following the “lyotropic” sequence SCN^−^>I^−^>Br^−^>Cl^−^>F^−^ [10]. Moreover the permeant anions differently affect the channel conductance and the apparent Ca^2+^-sensitivity [10].

A long lasting effort to find the molecular counterparts of CaCCs culminated in 2008 with the discovery of two members of the TMEM16 family, TMEM16A and TMEM16B (also known as anoctamin-1 and -2) [11–13]. The TMEM16 family is well conserved through the evolution and in vertebrates it is composed of ten members (TMEM16A to K with I skipped; [14]). Even if the function of some TMEM16 proteins has not been characterized yet, different studies showed a big functional variability. Indeed, TMEM16 can be an ion channel (A, B and F [11–13,15–17]), a regulator of other ion channels (C, [18]) or a scramblase (C, D, F, G and J; [19].

**Fig 1:**
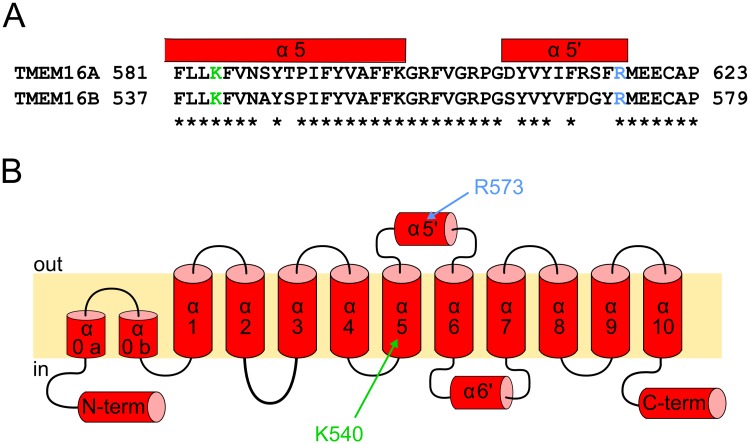
TMEM16 proposed topology. (A) Alignment between TMEM16A and TMEM16B with mutated residues highlighted in color. (B) Proposed topology of TMEM16B based on the structure of nhTMEM16 with the mutated amino acids indicated in their predicted positions.

In 2014, Brunner et al. [20] solved the crystal structure of a TMEM16 from the fungus *Nectria haematococca* named nhTMEM16. The closest mammal homologues of nhTMEM16 are TMEM16H and K. However, the CaCCs TMEM16A and B retain about 40% homology with the transmembrane region of nhTMEM16 suggesting that all members of the family share a similar structure [20]. Functional characterization of nhTMEM16 using reconstituted protein into liposomes showed that it could act as Ca^2+^-dependent scramblase mediating the transport of the phospholipids across the two membrane leaflets [20]. However, all attempts to detect any ion channel activity mediated by nhTMEM16 have—so far—failed [20]. The X-ray structure of nhTMEM16 showed that it formed a dimeric protein with a rhombus shape of about 130 X 40 Å in dimension when viewed from an extracellular side [20]. Both the N- and C- termini were localized on the intracellular side of the membrane and they were responsible for the largest part of the interface surface between the two dimer subunits [20]. Biochemical studies showed that also mouse TMEM16A, B and F formed homodimers [21–23] and with mutagenesis experiments in TMEM16A a short N-terminus region between residues 117 and 179 was found sufficient for dimer formation, necessary condition for proper channel trafficking to plasma membrane (for TMEM16A all the numbers refer to splice variant “a” as in [11]; [23]). The transmembrane region of nhTMEM16 is composed by 10 membrane-spanning α-helices (α1 to α10, Fig 1B) preceded by two short α-helices (α0a and α0b) that only peripherally interact with the inner membrane leaflet [20]. Moreover, two short α-helix, α5' and α6', are located respectively in the extracellular loop connecting α5 and α6 and intracellularly, in the loop between α6 and α7. The crystal structure revealed that each subunit organized a Ca^2+^-binding site composed by three glutamates (E452, E506, E535), two aspartates (D503, D539) and an asparagine (N448). All these residues are highly conserved in most TMEM16 proteins and mutagenesis studies showed that all the charged residues control the Ca^2+^-sensitivity in TMEM16A and F indicating that they share a Ca^2+^-activation mechanisms [17,24,25]. Moreover, these residues are localized in the transmembrane α-helices (α6, 7 and 8) explaining the voltage dependent Ca^2+^-sensitivity observed in TMEM16A, B and F [15–17,24]. While the nhTMEM16 structure did not show a clear pore resembling those of the voltage-gated cation channel [26], it showed a narrow twisted crevice 8–11 Å wide composed by the helices α3 to α7 of the same subunit facing the membrane and it has been proposed to be forming the channel pore [20]. Indeed, mutagenesis studies in the equivalent region of TMEM16A and F found that the residues facing this crevice control the ion selectivity [17,27,28] or the ion conductance [25] indicating a key role on ion transportation. However, studies on mouse TMEM16A reported contradictory results about the role of the residue R617. Based on the structure of nhTMEM16, R617 is located in the extracellular loop connecting α5 and α6, at the entrance of the protein crevice [20]. And while Yang et al. [13] reported that R617E mutant had an altered ion selectivity, a later study did not find significant differences with the wt channel [25]. Nevertheless, reports on R617A mutant found that there is an alteration in permeability properties when the channel is activated by high [Ca^2+^]_i_.

Here, I mutagenized the homologous of R617 in TMEM16B (R573, Fig 1) to clarify its role in controlling the ion selectivity; moreover, I studied the permeability of a mutant of the homologous of K584 (K540, Fig 1) located deeper in the putative channel pore.

## Materials and Methods

### Site-directed mutagenesis of TMEM16B and heterologous expression

Full-length mouse TMEM16B cDNA in pCMV-Sport6 mammalian expression plasmid was obtained from RZPD (NCBI Protein database accession NP_705817.1). Mutations were made using a PCR-based site directed mutagenesis and confirmed by DNA sequencing. HEK 293 (American Type Culture Collection) cells were transfected with 2 μg TMEM16B by using X-tremeGENE (Roche) following manufacturer instructions. Cells were cotransfected with 0.4 μg mCherry (Clontech) for fluorescent identification of transfected cells.

### Electrophysiological recordings and ionic solutions

Electrophysiological recordings were performed using the whole-cell and inside-out patch clamp configurations. Borosilicate glass pipettes (WPI) were pulled with a PP-830 puller (Narishige). Currents were recorded with an Axopatch1-D amplifier controlled by Clampex 9 via a Digidata 1332A (Axon Instruments). Data were sampled at 10 kHz after a low-pass filter at 4 kHz. Experiments were performed at room temperature (20–22°C). The bath solution was grounded via a 3M KCl agar bridge connected to an Ag/AgCl reference electrode. Fast-Step SF77B (Warner Instruments Corp.) were used to rapid (<5 ms) solutions exchanging. Liquid junction potentials were calculated by pClampex software and the applied voltages were corrected off-line.

For inside-out recordings, the standard solution in the patch pipette contained (in mM): 140 NaCl, 10 HEDTA, and 10 Hepes, pH 7.2. The bathing solution at the intracellular side of the patch contained (in mM): 140 NaCl, 10 HEDTA, and 10 Hepes, pH 7.2, and no added Ca^2+^ for the nominally 0 Ca^2+^ solution, or various added Ca^2+^ concentrations, as calculated with the program WinMAXC(C. Patton), to obtain free Ca^2+^ in the range between 1.5 and 100 μM. For the solution containing 1 mM of Ca^2+^ HEDTA was omitted. For experiments using N(CN)_2_^−^ and C(CN)_3_^−^ 100 μM free Ca^2+^ concentration was obtained directly by adding 100 μM of CaCl_2_ without HEDTA. Since C(CN)_3_^−^ was available only as potassium salt, symmetrical KCl solutions were used as control conditions. A previous report [29] showed that solutions containing SCN^−^ and C(CN)_3_^−^ can have different effects depending on the age of the solution, therefore we prepared new solutions weekly.

In some experiments NaCl in bath or pipette solutions was reduced to 100, 50, 20, 5 mM and sucrose was added to maintain the osmolality. For permeability experiments, Cl^−^ was substituted with other anions by replacing NaCl on an equimolar basis with NaX, where X is the substituted anion.

For whole-cell recordings, the cells were kept in mammalian Ringer standard solution containing (in mM): 140 NaCl, 5 KCl, 2 CaCl_2_, 1MgCl_2_, and 10 Hepes, pH 7.4. The pipette solution contained (in mM): 140 NaCl, 10 Hepes, and 10 HEDTA, pH 7.2, and Ca^2+^ solution, 1.242 or 3.209 mM CaCl_2_ to obtain 0.5 or 1.5 μM free Ca^2+^ respectively. Permeability experiments were performed bathing the cells in a solution containing (in mM): 140 NaCl, 10 Hepes, 10 HEDTA pH 7.2 or 140 NaSCN, 1 NaCl, 10 Hepes, 10 HEDTA pH 7.2.

### Data analysis

Data are presented as mean ± SEM (standard error of the mean), with *n* indicating the number of cells or excided patches. Since some data were not normally distributed (tested with Shapiro-Wink test) the statistical significance was determined using U-test (Wilcoxon-Mann-Whitney rank test) or Wilcoxon signed rank test for paired data, or the Kruskal Wallis test for multiple comparisons. When statistical significance was determined by Kruskal Wallis test, a post hoc Dunn test was performed to evaluate which data groups showed significant difference. *p* value <0.05 was considered significant. Data analysis and figures were made with Igor Pro Software (Wavemetrics).

## Results

### Cationic-anionic permeability in TMEM16B wt and mutant channels

**Fig 2:**
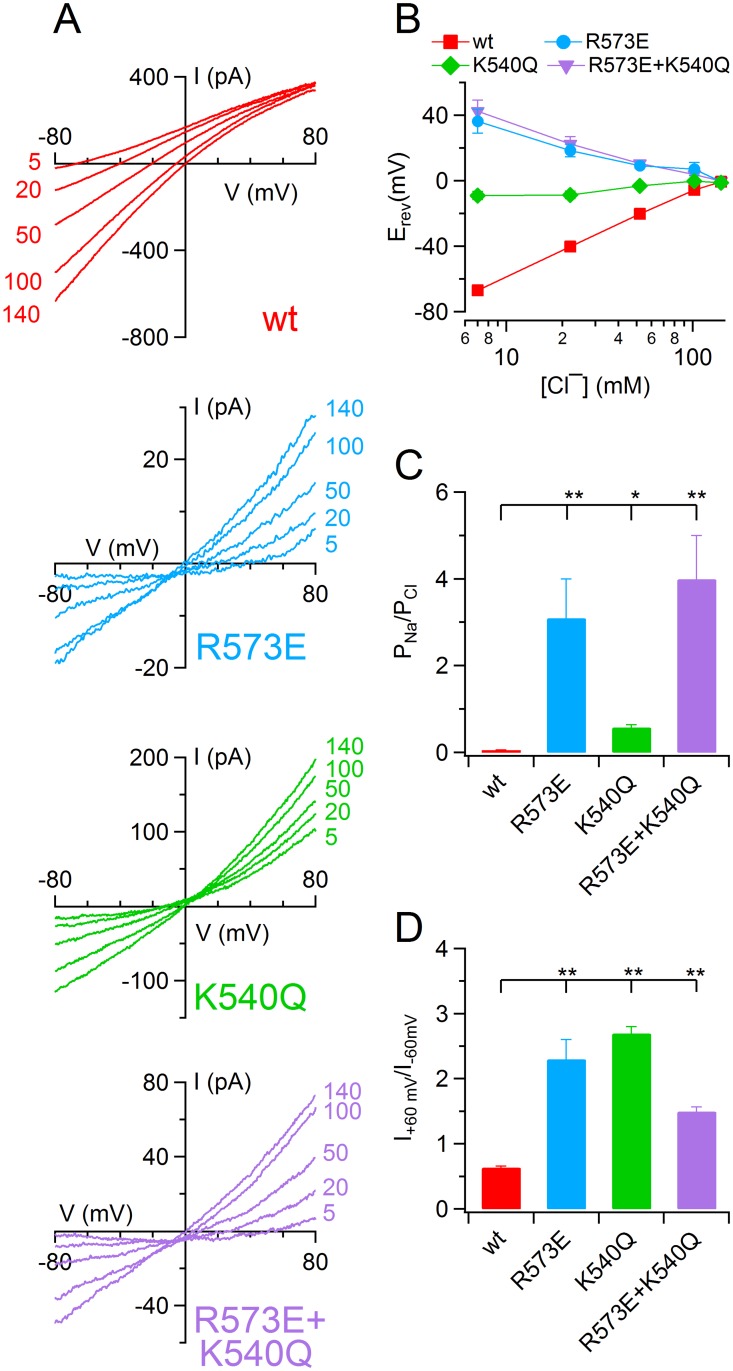
Intracellular sodium permeability in TMEM16B wt and mutant channels. An inside-out patch expressing TMEM16B wt or the indicated mutants was exposed to different cytoplasmatic [NaCl] and the IV relation was determined by a voltage ramp from -100 to +100 mV. The current was activated by 1 mM CaCl_2_. Leakage currents measured in 0 Ca^2+^ were subtracted. (B) Average E_rev_ corrected for liquid junction potential was plotted versus [Cl^−^]_i_ for TMEM16B wt and mutant channels (*n* = 5–6). (C) Mean permeability ratio between Na^+^ and Cl^−^ (P_Na_/P_Cl_) in TMEM16B wt and mutant channels calculated with Goldman-Hodgkin-Katz equation from recordings with 20 mM [NaCl]_i_ (*n* = 6). (D) Ratios between the currents measured at +60 and -60 mV with symmetrical 140 NaCl solutions for TMEM16B wt and mutant channels (*n* = 7–12; *p<0.05 **p<0.01 Dunn test after Kruskal Wallis test).

To study the ionic permeability of wt and mutated TMEM16B, I performed inside-out experiments from excised patches from HEK-293 cell transiently transfected. This approach allowed a good control of the solutions facing both sides of the membrane; therefore it reduced the possible effects due to local ion accumulation occurring in whole-cell recordings. Fig 2A, top panel, shows currents activated with 1 mM Ca^2+^ with voltage ramp protocol from +100 mV to -100 mV in the presence of the indicated concentration (in mM) of intracellular NaCl. The reduction of intracellular NaCl from 140 to 5 mM shifted the reversal potential from -0.5 ± 0.2 mV to -67 ± 2 mV (*n* = 7), indicating that TMEM16B mediated a current mainly carried by Cl^−^ ions. Indeed the permeability ratio between Na^+^ and Cl^−^ (P_Na_/P_Cl_) calculated by Goldman-Hodgkin-Katz equation resulted to be 0.051 ± 0.005 (*n* = 7). I repeated the same experiments using HEK-293T cells expressing R573E, K540Q and R573E+K540Q TMEM16B mutants. Fig 2A illustrates voltage ramp recordings from each mutant in the presence of different intracellular NaCl concentrations. In contrast with the wt channel, in R573E and R573E+K540Q mutants, the reduction of intracellular [NaCl] from 140 to 5 mM shifted the reversal potential toward positive values by 36 ± 7 (*n* = 5) and 42 ± 7 (*n* = 6) respectively (Fig 2B). This indicates a change in cationic versus anionic selectivity; indeed the P_Na_/P_Cl_ became 3.1 ± 0.9 for R573E and 4 ±1 (*n* = 6) for R573E+K540Q (Fig 2C). Whereas the K540Q mutant retained a preference toward anion permeability even though the sodium permeability significantly increased about 100 fold (Fig 2C). These results show that both R573 and K540 residues are important structural elements to control the permeation in the TMEM16B channel.

Interestingly, all TMEM16B mutants showed an altered rectification behavior. Indeed, while in TMEM16B wt the ratio between the current amplitude at +60 and -60 mV upon stimulation with saturating [Ca^2+^]_i_ was 0.64 ± 0.02 (*n* = 11) indicating an inward rectification, in the mutants it ranged from 1.51 ± 0.07 (*n* = 12) for R573E+K540Q to 2.7 ± 0.1 (*n* = 7) for K540Q indicating an outward rectification (Fig 2D).

**Fig 3:**
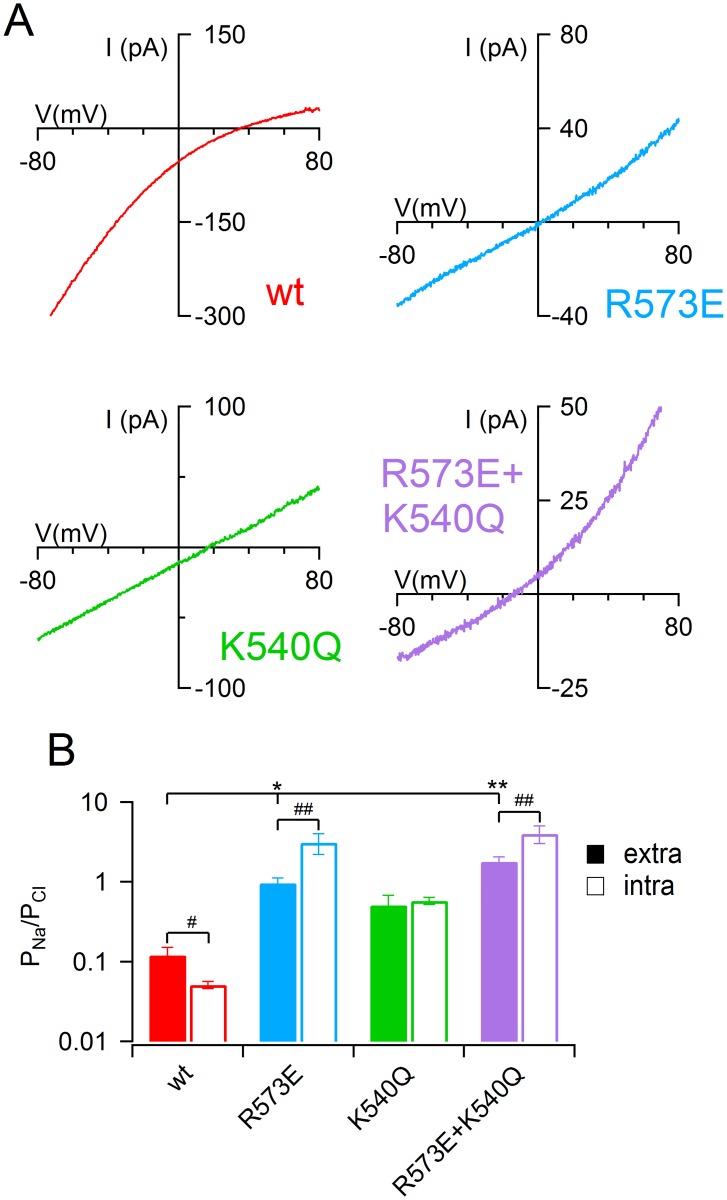
Extracellular sodium permeability in TMEM16B wt and mutant channels. (A) An inside-out patch expressing TMEM16B wt or the indicated mutant was exposed to bath solution with 140 mM NaCl while the pipette solution contained only 20 mM of NaCl. The IV relation was determined by a voltage ramp from -100 to +100 mV and the current was activated by 1 mM CaCl_2_. Leakage currents measured in 0 Ca^2+^ were subtracted. (B) Mean permeability ratio between Na^+^ and Cl^−^ (P_Na_/P_Cl_) in TMEM16B wt and mutant channels calculated with Goldman-Hodgkin-Katz equation from recordings with 20 mM NaCl in the pipette or in the bath solution (*n* = 6–11; *p<0.05 **p<0.01 Dunn test after Kruskal Wallis test; #p<0.05 ##p<0.01 U-test).

To test possible asymmetric cationic vs anionic permeation through the TMEM16B pore, I also evaluated the ionic permeability reducing the NaCl concentration to 20 mM in the pipette solutions bathing the extracellular leaflet of the membrane. Fig 3A shows the current activated by 1 mM of Ca^2+^ with a ramp protocol from +100 to -100 mV. The reversal potential in these ionic conditions for TMEM16B wt channel was 35 ± 3 mV (*n* = 7), significantly different from -0.5 ± 0.2 obtained in symmetrical NaCl solutions (*n* = 7, U-test, Fig 2B). Interestingly, these experiments indicated that the Na^+^ permeability was significantly higher than the value obtained by reducing the intracellular NaCl concentration, indeed P_Na_/P_Cl_ changed from 0.051 ± 0.05 to 0.12 ± 0.03 (U- test), indicating an asymmetrical cation permeation in TMEM16B wt channel. Similar to the results obtained by reducing the intracellular [NaCl] (Fig 2), R573E single and R573E+K540Q double mutants showed a significant increase of Na^+^ permeability. However, in contrast with the wt channel, in both mutants P_Na_/P_Cl_ was lower for the permeation from the extracellular side. Finally, K540Q mutants showed a symmetrical P_Na_/P_Cl_ and the permeation from the extracellular side was not significantly different from TMEM16B wt channel.

**Fig 4:**
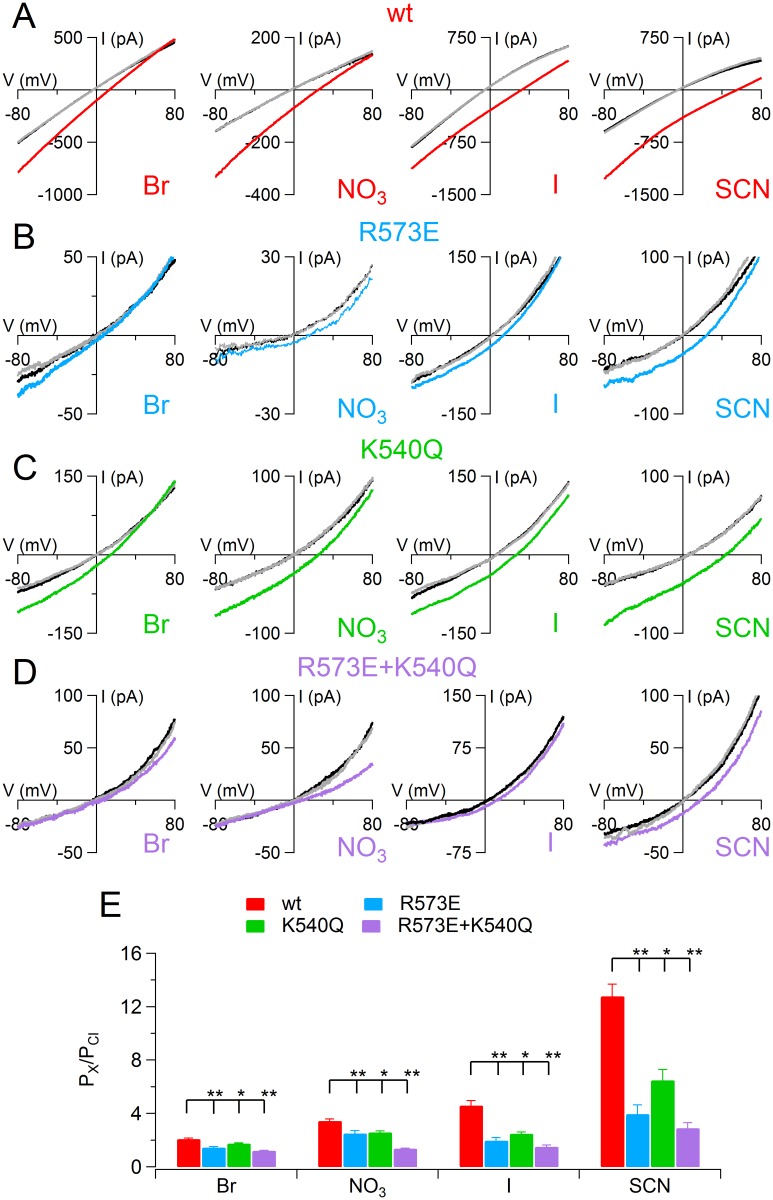
Anionic selectivity in TMEM16B wt and mutant channels. An inside-out patch expressing TMEM16B wt (A), R573E (B), K540Q (C), or R573E+K540Q (D) was exposed to bath solutions containing 140 mM NaCl (black traces) or the Na salt of other anions, as indicated. The gray traces represent the wash out with NaCl. The IV relation was determined by a voltage ramp from -100 to +100 mV and the current was activated by 100 μM [Ca^2+^]_i_. Leakage currents measured in 0 Ca^2+^ were subtracted. (E) Average permeability ratio between substituted anions and Cl^−^ (P_X_/P_Cl_) calculated with Goldman-Hodgkin-Katz equation (*n* = 3–10; *p<0.05 **p<0.01 Dunn test after Kruskal Wallis test).

In another set of experiments, I determined the anionic selectivity of TMEM16B mutants. I replaced the NaCl in the bath solution with equimolar NaBr, NaI, NaNO_3_ and NaSCN and I activated the current with 100 μM Ca^2+^ using a ramp from +100 to -100 mV. As previously reported TMEM16B wt channel was more permeable to anions bigger than Cl^−^[15,16,30]. Relative permeability ratios (P_X_/P_Cl_) were calculated with Goldman-Hodgkin-Katz equation and followed the sequence SCN (14) > I (4.6) > NO_3_ (3.5) > Br (2.08) (Fig 4A–4E). Also in all mutants Br^−^, NO_3_^−^, I^−^, SCN^−^ were more permeable than Cl^−^ but the relative permeability ratios were significantly reduced (Fig 4B–4E). For example, P_I_/P_Cl_ was 2.0 ± 0.2 for R573E, 2.4 ± 0.1 for K540Q and 1.5 ± 0.1 for R573E+K540Q, significantly smaller than 4.6 ± 0.4 measured in wt channel (*n* = 6–10, Dunn-test after Kruskal Wallis test). Moreover, R573E mutants showed also an alteration in the permeability sequence with respect to the wt channel; indeed I^−^ was less permeable than NO_3_^−^.

**Fig 5:**
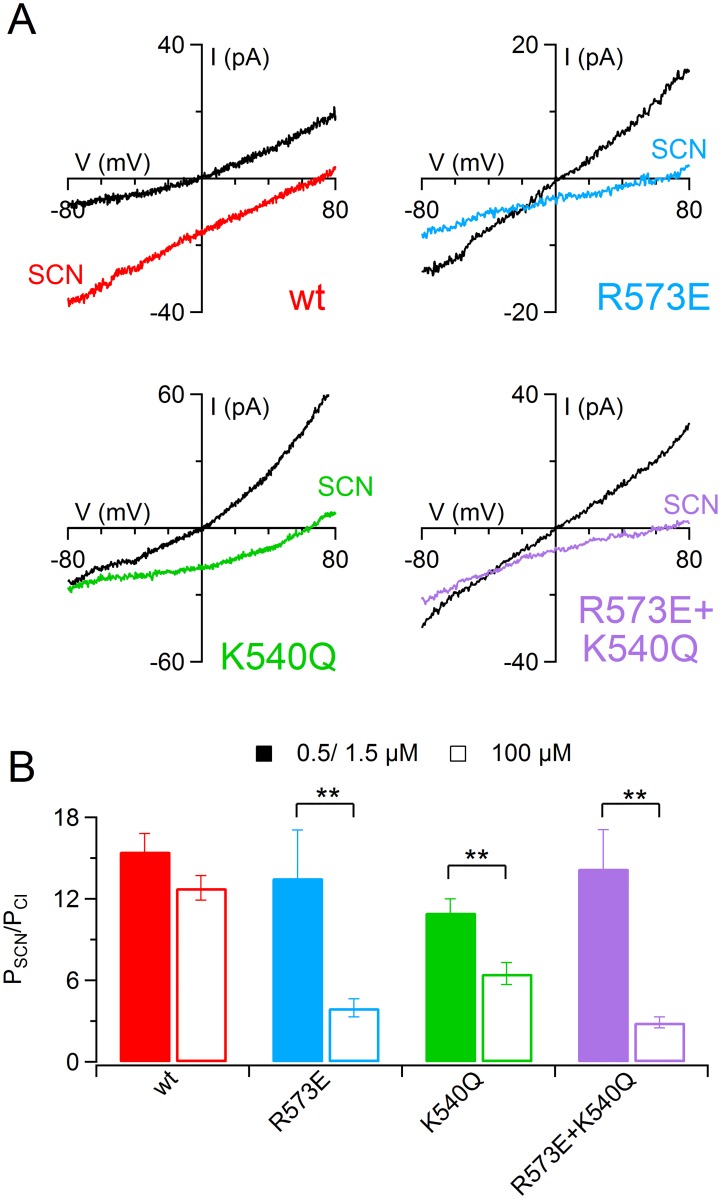
SCN^−^ permeability in TMEM16B wt and mutant channels at low [Ca^2+^]_i_. (A) An inside-out patch expressing TMEM16B wt or the indicated pore mutants was exposed to bath solution containing 140 mM NaCl concentrations (black traces) or NaSCN (colored traces). The IV relation was determined by a voltage ramp from -100 to +100 mV and currents were activated by 0.5 μM [Ca^2+^]_i_ for wt and K540Q, or 1.5 μM [Ca^2+^]_i_ for R573E and R573E+K540Q. Leakage currents measured in 0 Ca^2+^ were subtracted. (B) Mean permeability ratio between SCN^−^ and Cl^−^ (P_SCN_/P_Cl_) for TMEM16B wt and mutant channels with 100 μM [Ca^2+^]_i_ (empty bars, data from Fig 4E) or sub-saturating [Ca^2+^]_i_ (filled bars. *n* = 6–14; ** p<0.01 U-test).

Finally, since a previous report showed that in TMEM16A anion permeability was affected by the level of channel activation by [Ca^2+^]_i_ [27], I determined the permeability of SCN^−^ of wt and of mutant TMEM16B channel (Fig 5A) upon stimulation of sub-saturating [Ca^2+^]_i_ of 0.5 μM (for wt and K540Q), or 1.5 μM (for R573E and R573E+K540Q). A comparison among P_SCN_/P_Cl_ of wt and mutant TMEM16B channel at sub-saturating [Ca^2+^]_i_ (Fig 5B) did not show any significant difference (p>0.05, Kruskal Wallis test). In addition, P_SCN_/P_Cl_ for wt TMEM16B channel was not affected by [Ca^2+^]_i_ (12.8 ± 0.9 at 100 μM vs 15 ± 1 at 0.5 μM, n = 6–14, p>0.05 U-test), whereas P_SCN_/P_Cl_ for each mutant was significantly higher at sub-saturating than at 100 μM [Ca^2+^]_i_. For example, P_SCN_/P_Cl_ for K540Q was 6.4 ± 0.8 at 100 μM vs 11 ± 1 at 0.5 μM [Ca^2+^] (n = 6, p<0.05 U-test). Moreover, similar results were obtained activating wt and K540Q with 1.5 μM instead of 0.5 μM [Ca^2+^]_i_ (data not shown). These results show that R573E and K540Q residues in TMEM16B channel modify anion permeability only at high [Ca^2+^]_i_.

### Ca^2+^-sensitivity of TMEM16B pore mutants

**Fig 6:**
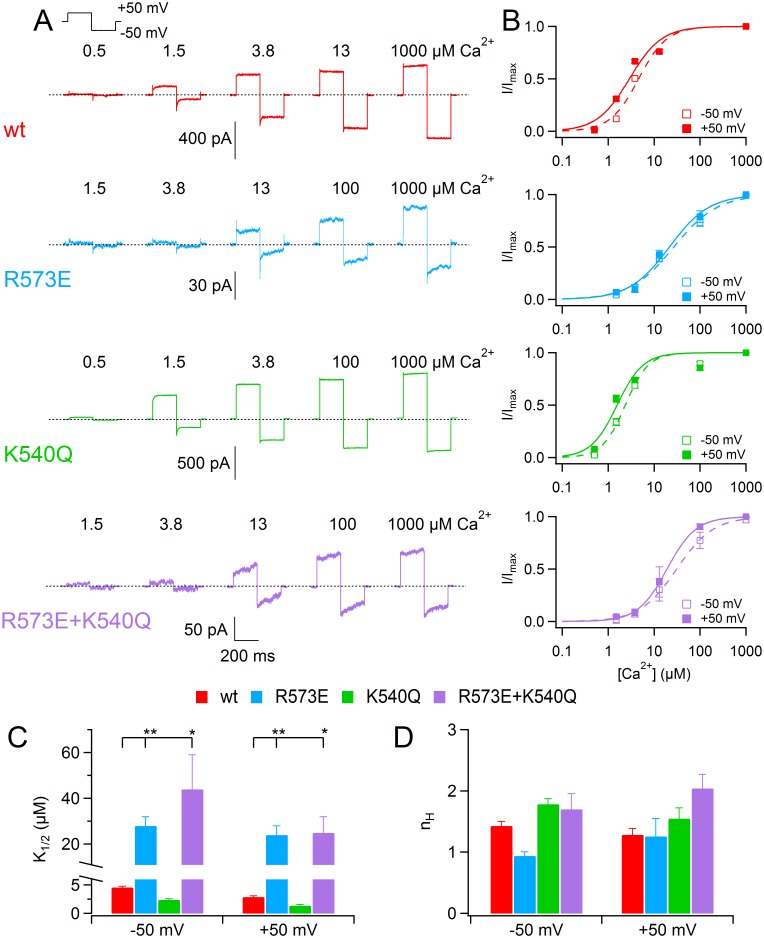
Ca^2+^ sensitivity of TMEM16B wt and mutant channels. (A) Each row shows current traces from the same inside-out patch expressing TMEM16A wt or the indicated mutants. The cytoplasmatic side was exposed to [Ca^2+^]_i_ ranging from 0.5 to 1000 μM. Voltage steps of 200 ms duration were given from the holding voltage of 0 mV to +50 mV, followed by 200 ms step to -50 mV as indicated in the top part. Leakage currents measured in 0 Ca^2+^ were subtracted. (B) Dose-response relations of activation by Ca^2+^ were obtained by normalized currents at -50 and +50 mV and fitted with Hill equation (*n* = 6–7). (C) Mean of K_1/2_ at -50 and +50 mV for TMEM16B wt and mutant channels (*p<0.05 **p<0.01 Dunn test after Kruskal Wallis test). (D) Mean of n_H_ at -50 and +50 mV for TMEM16B wt and mutant channels (p>0.05 Kruskal Wallis test).

Since previous reports showed a dependence of calcium sensitivity by permeating ions [29,31], I examined the dependence of TMEM16B pore mutants on intracellular Ca^2+^ concentration under symmetrical NaCl conditions. I activated the current by various [Ca^2+^]_i_ and stepped the membrane voltage to -50 and +50 mV as shown in Fig 6A. I measured the current amplitude at the end of each voltage step by taking the mean value between 175 and 195 ms, normalized to the maximum value at the same voltage and plotted versus the [Ca^2+^]_i_ (Fig 6B). Data were fitted by Hill equation:

$$\frac{I}{I_{max}}=\frac{{\left[Ca^{2+}\right]}_{i}^{n_{H}}}{{\left[Ca^{2+}\right]}_{i}^{n_{H}}+K_{1/2}^{n_{H}}}$$ (1)

where *I* is the current, *I*_*max*_ is the maximum current, K_1/2_ is the half-maximum [Ca^2+^]_i_, and n_H_ is the Hill coefficient.

As previously reported [15,16,32], in TMEM16B wt channel the K_1/2_ was slightly voltage-dependent ranging from 4.6 ± 0.2 μM at -50 mV to 2.9 ± 0.1 μM at +50 mV (*n* = 7; Wilcoxon signed rank test; Fig 6C). Similar results were obtained for K540Q mutant, whereas R573E and R573E+K540Q showed a significant increase of K_1/2_ reaching at -50 mV 28 ± 4 μM and 44 ± 15 μM respectively. Moreover, in R573E mutant there was no significant difference of apparent Ca^2+^-sensitivity between positive and negative potentials (Wilcoxon signed rank test). For all mutants n_H_ was between 1 and 2 and it was not significantly different from the wt value (Fig 6D). These data suggest a possible molecular coupling between channel permeation and gating.

### Anomalous mole fraction effect

**Fig 7:**
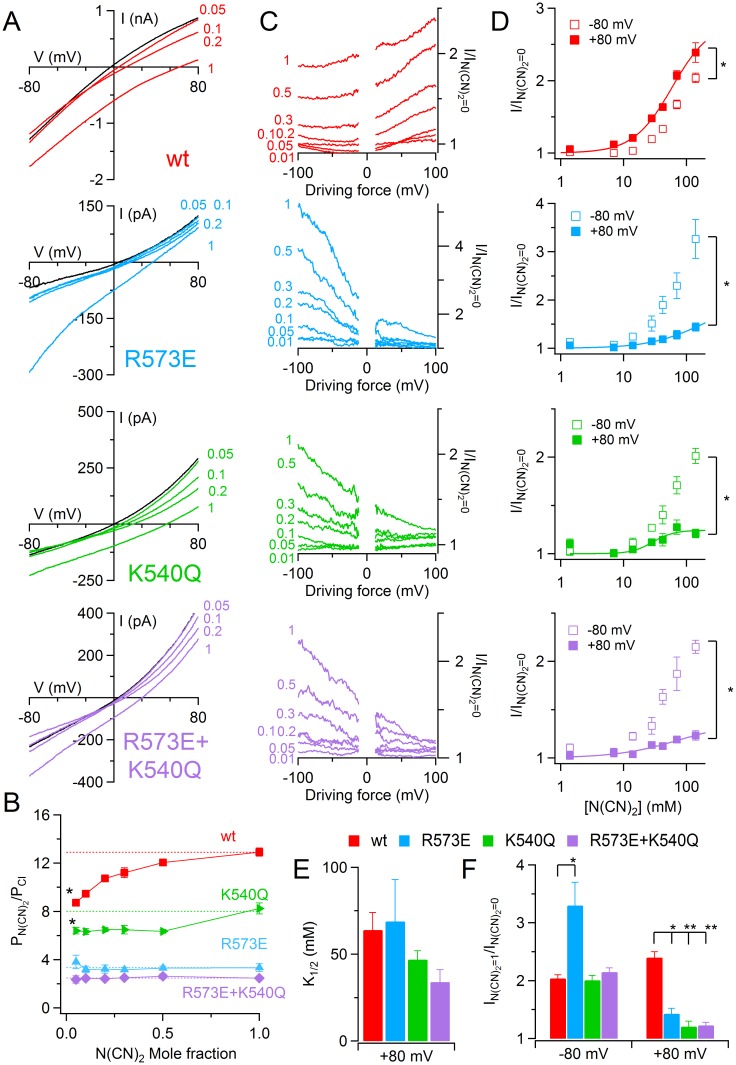
Anomalous mole fraction behavior of TMEM16B wt and mutant channels. (A) An inside-out patch expressing TMEM16B wt or the indicated pore mutants was exposed to bath solution containing 140 mM NaCl concentrations (black traces) or the indicated mole fraction of NaN(CN)_2_ (colored traces). The IV relation was determined by voltage ramp from +100 to -100 mV and the currents were activated by 100 μM [Ca^2+^]_i_. Leakage currents measured in 0 Ca^2+^ were subtracted. (B) Mean permeability ratio between N(CN)_2_^−^ and Cl^−^ (P_N(CN)2_/P_Cl_) was plotted versus the mole fraction of the substituted anion (*n* = 6–15). The dotted line highlighted the permeability ratio with mole fraction equal to 1 (*p<0.05 Wilcoxon signed rank test). (C) Ratios between the current amplitude in the presence of the indicated N(CN)_2_^−^ intracellular concentration with symmetrical Cl^−^ solution (I/I _N(CN)2 = 0_) calculated from experiments in (A) were plotted versus the driving force. (D) Mean of the I/I _N(CN)2 = 0_ measured at +80 and -80 mV of the driving force was plotted versus the N(CN)_2_^−^ intracellular concentration. The continuous line represents the fit with Hill equation (Eq 1; *n* = 5–6; *p<0.05 Wilcoxon signed rank test between the values at -80 and +80 mV for 140 mM N(CN)_2_^−^). (E) Mean of K_1/2_ at +80 mV of the driving force for TMEM16B wt and mutant channels. (F) Mean of the ratios between the current measured with a solution containing 140 mM N(CN)_2_^−^, mole fraction 1, and in control conditions, mole fraction 0, at -80 and +80 mV of the driving force for TMEM16B wt and mutant channels (**p<0.01, *p<0.05 Dunn-test after Kruskal Wallis test).

Previous reports showed that TMEM16A and some native Ca^2+^-activated Cl^−^ channels [28,29] have an anomalous mole fraction effect in the presence of more permeable anions. Therefore, I tested the permeability of TMEM16B in the presence of different amounts of SCN^−^ and dicyanamide N(CN)_2_^−^ upon stimulation of the current with saturating Ca^2+^ concentration in excised inside-out patches. As previously shown for native Ca^2+^-activated Cl^−^ channels of *Xenopus* oocytes, I did not find a dependence of SCN^−^ permeability on mole fraction (data not shown, [29]). In contrast, I observed that relative permeability ratio between N(CN)_2_^−^ and Cl^−^ (P_N(CN)2_/P_Cl_) changed based on dicyanamide mole fraction. In particular, P_N(CN)2_/P_Cl_ increased with the raising of N(CN)_2_^−^ intracellular concentration ranging from 8.7 ± 0.3 to 13 ± 0.9 for 0.05 and 1 mole fraction respectively (*n* = 6; Wilcoxon signed rank test, Fig 7A–7D). All pore mutants showed a significant reduction of the dicyanamide permeation confirming their role in the control of the ion pathway (Fig 7D). Moreover, interestingly, both single R573E and R573E+K540Q double mutant did not show an anomalous mole fraction behavior, indeed P_N(CN)2_/P_Cl_ did not significantly change from 0.05 to 1 mole fraction (Fig 7A–7D, Wilcoxon signed rank test). In contrast, K540Q mutant retained dicyanamide permeation dependence on anion concentration in intracellular solution (Fig 7A–7D, Wilcoxon signed rank test).

Then I investigated the effect of N(CN)_2_^−^ on the current amplitude. Therefore I plotted the ratio between the current in the presence of different amount of intracellular N(CN)_2_^−^ and in control conditions (I/I _N(CN)2 = 0_) versus the driving force (V-E_rev_; Fig 7C). Fig 7D shows the current ratios at +80 and -80 mV of the driving force plotted versus the intracellular N(CN)_2_^−^ concentration. In TMEM16B wt channel N(CN)_2_^−^ induced a voltage dependent increase of current amplitude that was higher at +80 than at -80 mV of the driving force. The data at +80 mV could be fitted by Hill equation and gave a K_1/2_ of 65 ± 10 mM (*n* = 6, Fig 7D and 7E). In all pore mutants N(CN)_2_^−^ also induced a voltage dependent change of the current amplitude but in the opposite direction, indeed the increase induced by N(CN)_2_^−^ at -80 mV of the driving force was significantly higher than at +80 mV (Fig 7D; Wilcoxon signed rank test). Moreover, while at -80 mV only R573E mutants showed a significant higher effect of N(CN)_2_^−^ on current amplitude with respect to the wt channel, at +80 mV of the driving force all pore mutants showed a reduction of N(CN)_2_^−^ effect (Fig 7F). However, the K_1/2_ obtained by fitting the data at +80 mV with Hill equation (Eq 1) did not show a significant change from the values obtained from TMEM16B wt channel.

### Blockage by permeant anions

**Fig 8:**
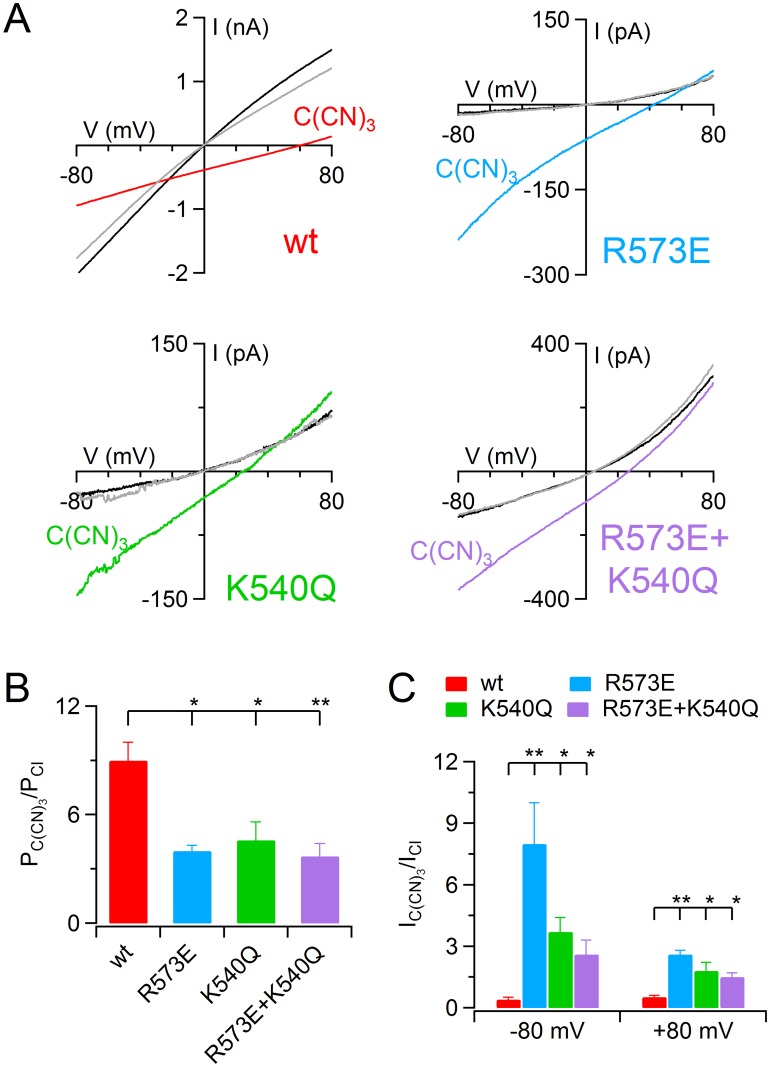
Activation by C(CN)_3_^−^ in TMEM16B pore mutants. (A) An inside-out patch expressing TMEM16B wt or the indicated pore mutants was exposed to bath solution containing 140 mM KCl concentrations (black traces) or the indicated mole fraction of KC(CN)_3_ (colored traces). The gray traces represent the wash out with KCl. The IV relation was determined by a voltage ramp from -100 to +100 mV and the current was activated by 100 μM [Ca^2+^]_i_. Leakage currents measured in 0 Ca^2+^ were subtracted. (B) Mean permeability ratio between C(CN)_3_^−^ and Cl^−^ (P_C(CN)3_/P_Cl_) for TMEM16B wt and mutant channels calculated with Goldman-Hodgkin-Katz equation. (C) Mean of the ratios between the current measured with a solution containing 140 mM C(CN)_3_^−^ and in control conditions, at -80 and +80 mV of the driving force for TMEM16B wt and mutant channels (**p<0.01, *p<0.05 Dunn-test after Kruskal Wallis test).

Previous investigations on native Ca^2+^-activated Cl^−^ channel of *Xenopus* oocyte showed that some permeant anions could block the channel [29]. Therefore, I investigated the effect of tricyanomethanide, C(CN)_3_^−^ on TMEM16B channel. I measured the currents in excised inside-out patches replacing the KCl bath solution with KC(CN)_3_. The currents were activated by 100 μM Ca^2+^ using voltage ramp from +100 to -100 mV. Replacement of Cl^−^ with C(CN)_3_^−^ shifted the reversal potential toward positive values indicating a bigger relative permeability with respect to Cl^−^. Indeed, P_C(CN)3_/P_Cl_ calculated with Goldman-Hodgkin-Katz equation resulted 9 ± 1 (*n* = 6, Fig 8A and 8B). To evaluate the effect of tricyanomethanide on the conductance I calculated the ratio between the current amplitude in the presence of C(CN)_3_^−^ and in Cl^−^ symmetrical conditions at -80 and +80 mV of driving force. At both driving forces C(CN)_3_^−^ induced a significant reduction of the current, indeed I_C(CN)3_/I_Cl_ reached 0.4 ± 0.1 at -80 mV and 0.52 ± 0.08 at +80 mV (*n* = 6, Wilcoxon signed rank test, Fig 7C). I repeated the same experiments with pore mutants and, similar to the results obtained for the other anions (Fig 4), C(CN)_3_^−^ was found to be less permeable in mutants than in TMEM16B wt channel (Fig 8A and 8B). Moreover, in contrast with the wt channel, in all mutants tricyanomethanide did not cause a blockage of the channel but it induced a significantly increase of the current amplitude with a higher effect at -80 mV than +80 mV of driving force (Fig 8C). The activation of the current induced by C(CN)_3_^−^ was larger in R573E single mutants with an increase of 8 ± 2 and 2.6 ± 0.2 fold at -80 mV and +80 mV of the driving force respectively (Fig 8C).

### Extracellular anionic selectivity in whole-cell recordings

**Fig 9:**
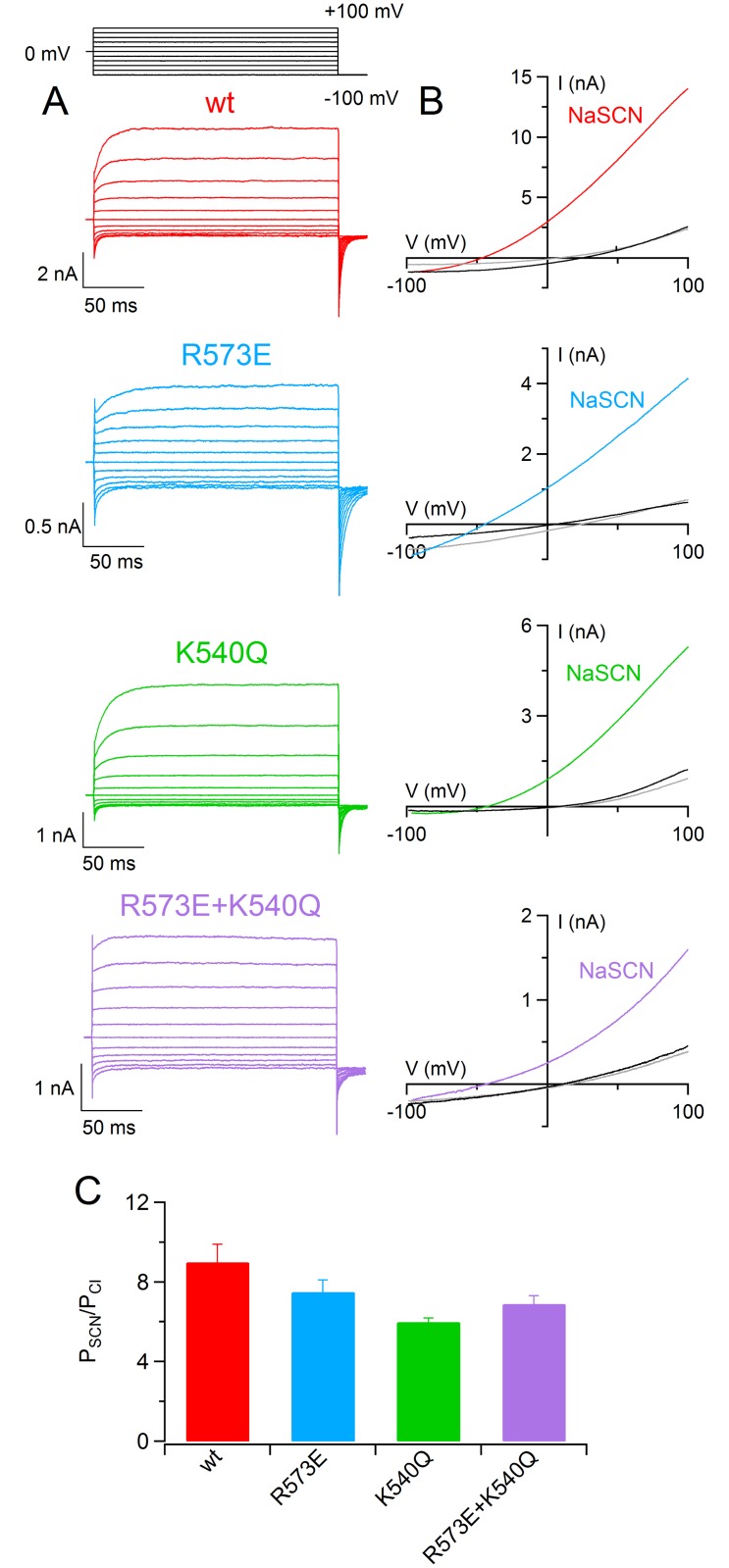
Extracellular ion selectivity in whole-cell recordings. (A) Each row shows representative whole-cell recordings obtained from HEK-293 cells expressing TMEM16 wt or the indicated mutants. The currents were activated with 0.5 μM [Ca^2+^]_i_ (TMEM16 wt and K540Q) or with 1.5 μM [Ca^2+^]_i_ (R573E and R573E+K540Q). Voltage steps of 200 ms duration were given from a holding potential of 0 mV to a voltage ranging from -100 to +100 mV followed by a step at -100 mV, as indicated in the top part of the panel. (B) IV relations measured by a voltage ramp from +100 to -100 mV from HEK-293 cells expressing TMEM16B wt or the indicated mutants in control extracellular solution containing NaCl (black traces), NaSCN (colored traces) and after wash out (gray traces). (C) Mean permeability ratio between SCN^−^ and Cl^−^ for TMEM16B wt and mutant channels (*n* = 5–7, p>0.05 Kruskal Wallis test).

Finally, I measured the anionic permeability of TMEM16B channel in whole-cell recordings. I dialyzed the cells with a solution containing 0.5 μM (for wt and K540Q) or 1.5 μM (for R573E and R573E+K540Q) free Ca^2+^ concentration and I stimulated the current with a ramp protocol from +100 to -100 mV replacing NaCl from extracellular solution with NaSCN. In TMEM16B wt channel the replacement of NaCl with NaSCN caused a significant shift of reversal potential toward negative values. This indicated that SCN^−^ was more permeable than Cl^−^ ions. I calculated the relative permeability ratio between SCN^−^ and Cl^−^ using the Goldman-Hodgkin-Katz equation and obtained a P_SCN_/P_Cl_ of 9 ± 1 (*n* = 7), slightly but significantly lower than the value acquired in inside-out experiments (Figs 9 and 5, p<0.05 U-test). In accordance with the data obtained in inside-out recording using sub-saturating [Ca^2+^]_i_, P_SCN_/P_Cl_ for all mutants were not significantly different from the value obtained from TMEM16B wt channel (p>0.05 Kruskal Wallis test). These results confirmed the role of the level of channel activation in controlling mutant channels permeability (see Discussion).

## Discussion

Here, I provide a site-direct mutagenesis study investigating the molecular mechanisms of ion selectivity in the TMEM16B Ca^2+^-activated Cl^−^ channels. I mutated two residues, R573 and K540, both conserved between TMEM16A and B (Fig 1) and I found specific alterations in the ion selectivity, calcium sensitivity and the blockage by permeant anions. Based on crystal structure of nhTMEM16 [20], R573 is located at the entrance of the proposed pore of the protein in the extracellular loop between the transmembrane helices α5 and α6, while K540 is placed in the α5 inside of the putative pore. I performed experiments using both inside-out and whole-cell configurations. Both techniques have advantages and disadvantages. In particular, the inside-out configuration allows for a good control of the intracellular solution composition and to test several of them on the same patch. However, as previously reported [15,16,31,32] the current mediated by TMEM16B undergoes an irreversible rundown, therefore I performed the experiments in a time window in which the current amplitude was stable. Moreover, when using the inside-out configuration, it is far from trivial to change the solution facing the extracellular side of the membrane, which, on the contrary, can be easily obtained in whole-cell recordings. However, ion depletion or accumulation could affect the value of the reversal potential recorded in these conditions, especially in cells with high current amplitude. Therefore, I used inside-out recordings to assess the ion selectivity and the blockage by permeant anions in fully activated channels and to measure the Ca^2+^-sensitivity and the whole-cell recording to evaluate the ion permeation in only partially activated channels.

### Ion selectivity in R573 and K540 mutants

Inside-out experiments activating the channel with saturating [Ca^2+^]_i_ (1 mM or 0.1 mM) showed the single R573 and K540 or double mutant had an altered ion selectivity upon ion substitution from the intracellular side of the membrane. In particular, permeability to Na^+^ highly increased, while anions bigger than Cl^−^ (Br^−^, I^−^, NO_3_^−^ and SCN^−^) were less permeable than in the TMEM16B wt channel. These data strongly indicate that both R573 and K540Q are involved in controlling the ion permeation, possibly facing the ion pathway through the pore. The effects of R573E mutation was quantitatively bigger than those of K540Q and it could be explained by the charge inversion in R573E while in K540Q the positive charge has been only neutralized. On the other hand, since the anion permeability through TMEM16 channels depends monotonically (at least for the anions tested here) on the free energy of hydration [5,11–13,16], it is possible to speculate that R573 residue, located at the entrance of the pore, plays a prominent role in anion dehydration and in the selection of the permeant ion. Whereas K540, deep in the channel pore interacts slightly with the ions and therefore, its mutation causes the observed minor effect. Moreover, the R573 mutant showed an alteration in the anion permeability sequence, indeed NO_3_^−^ was more permeable than I^−^. This result is consistent with a recent report showing that the small pore of CFTR restricts the permeation of a large spherical ion such as I^−^ [33]. Indeed, if R573E mutation caused a unilateral or elliptical decrease of ion pathway this can explain both a reduction of the I^−^ permeability and the abolishment of anomalous mole fraction behaviors (see also later).

Inside-out recordings also revealed that wt TMEM16B channels had an asymmetrical Na^+^ permeability when fully activated, indeed P_Na_/P_Cl_ was 0.051 from intracellular side of the membrane, but it increased to 0.12 extracellularly. In contrast, our previous results showed no asymmetrical anionic permeation in TMEM16B channels [31] and similar findings were reported for Ca^2+^-activated Cl^−^ current in *Xenopus* oocytes [29], that are mediated by TMEM16A [12]. However, Qu and Hartzell did not test if Na^+^ permeability in TMEM16A was symmetrical [29]. Here I showed that mutation of R573 increased the extracellular Na^+^ permeability indicating that this residue controlled the movement of Na^+^ in both directions whereas K540 was not involved in the exclusion of Na^+^ translocation.

Surprisingly, in contrast with the results obtained at saturating [Ca^2+^]_i_, both inside-out and whole cell recordings did not show significant differences in ionic selectivity between TMEM16B wt and mutant channels when activated by sub-saturating [Ca^2+^]_i_. In accordance with my results the mutation of homologous of R573, R617 in TMEM16A did not show an altered P_Na_/P_Cl_ when activated with low Ca^2+^ in the patch pipette [25]. The dependence on the level of channel activation could also explain the contrasting results previously obtained by Yang et al. [13] on the same R617E mutant, showing an increase of P_Na_/P_Cl_ and a decrease of permeability to anions bigger than Cl^−^. Indeed, even if the precise [Ca^2+^]_i_ used to activate the R617E mutant in that report has not been measured, from the linear IV relation (see supplementary Fig 4 of [13]) it was expected to be high enough to fully activate the channel. It is possible to speculate that R573E and K540Q mutation unmasked the differential role of some residues in controlling the ion selectivity of TMEM16B depending on the level of the channel activation, however further experiments will be necessary to fully clarify this phenomenon.

Very recent studies [34,35] tried to alter the P_Cl_/P_Na_ permeability of TMEM16A as done by Yang et al., (2014, [17]) mutating the K584 (or K588) residue in TMEM16A (homologous to K540 in TMEM16B). Jeng et al. (2016, [35]) generated the K584Q mutation in TMEM16A and showed that K584 is located in the pore but could not measure any change of P_Cl_/P_Na_ caused by the K584Q mutation when channels were activated by 20 μM Ca^2+^. Lim et al. (2016, [34]) mutated the lysine into glutamate (K588E) but, because of the low magnitude of the Ca^2+^-activated currents, were not able to reliably characterize the ion selectivity of the mutant. However, the double mutant K588E/E702Q showed robust currents and P_Cl_ /P_Na_ did not vary in K588E/E702Q. In addition, they showed that P_Cl_ /P_Na_ in the wt TMEM16A did not change when Ca^2+^ was varied from 2 μM to 1 mM, although they did not report similar experiments for the double mutant. The reasons for the differences between results obtained by Lim et al. and Jeng et al. (2016, [34,35]) and those reported in Yang et al. (2012, [17]) are not clear, but Jeng et al. (2016, [35]) suggested that “differences in experimental methods, for example, using NMDG-SO_4_ versus sucrose to replace intracellular NaCl [34] and/or analyses (such as subtraction of the endogenous leak current; Fig S2 of [35]) may explain the discrepancy”.

Data on K540Q of TMEM16B presented in this paper showed not only a change of P_Na_ /P_Cl_ permeability (Fig 2) with respect to wt, but also changes in the permeability ratio of several anions compared to Cl^−^ at high Ca^2+^ concentrations (Fig 4). Differences between my results on TMEM16B and recent data on TMEM16A [34,35] could be due to differences among channels, or could be due to differences in experimental methods, such as the use of sucrose versus NMDG-SO_4_ to replace intracellular NaCl, but not to data analysis, as I did subtract the endogenous leak current in 0 Ca^2+^. Furthermore, the two recent studies [34,35] did not investigate permeability among anions and therefore I cannot further compare the results of my experiments obtained with different anions.

### Ca^2+^-sensitivity of TMEM16B pore mutants

Structural, biochemical and mutagenesis experiments showed that TMEM16 protein were activated by direct Ca^2+^-binding [20,24,25,36,37]. Both TMEM16A and B were activated in sub-micromolar/micromolar concentration range with TMEM16B slightly less sensitive than TMEM16A [15,16,24,30,32,38]. Moreover our previous results showed that in TMEM16B the apparent Ca^2+^ sensitivity depends on permeant anions [31]. For example, intracellular or extracellular substitution of Cl^−^ with SCN^−^ shifts the K_1/2_ at +100 mV from 2.8 μM to 1.28 μM and 0.8 μM respectively [31]. Also experiments on TMEM16A from *Xenopus tropicalis* reported an increase of Ca^2+^-sensitivity upon extracellular substitution of Cl^−^ with I^−^ [28], confirming the data from native Ca^2+^-activated Cl^−^ channel expressed in *Xenopus laevis* oocytes [29]. All these data indicate a connection between channel gating and ion permeation that seems a common feature of Cl^−^ channels, and in fact it has been observed also in ClC family [39].

Here I report that R573 mutation, which affected the Cl^−^ permeability of the channel, also caused a big decrease of apparent Ca^2+^-sensitivity. Given the positive charge of this residue and previous structural data and mutagenesis, it is unlikely that the observed results were due to a loss of direct interaction with Ca^2+^ ions in the mutated channel. It is instead tempting to speculate that R573E mutation affecting the binding of Cl^−^ allosterically alters the Ca^2+^ binding to the channel. Regardless of the precise molecular mechanisms, these data confirm a connection between gating and permeation in the TMEM16B channel.

### Anomalous mole fraction effect

Anomalous mole fraction effect (AMFE) was found in many different types of ion channels such as the voltage gated Ca^2+^-channel [40], the ryanodine receptor [41] or the cyclic nucleotide-gated channel [42] and also the CaCC expressed in *Xenopus* oocyte [29]. Moreover, studies on mouse and *Xenopus tropicalis* TMEM16A showed a significant deviation of reversal potential values recorded with different mixtures of Cl^−^ and other anions from the prediction obtained with the Goldman-Hodgkin-Katz equation [28,43].

Also here we find that TMEM16B shows an AMFE with dicyanamide: in our experiments, in fact, the P_N(CN)2_/P_Cl_ changed depending of N(CN)_2_^−^ mole fraction. Interestingly the AMFE was lost in R573E single and R573E+K540Q double mutants confirming the role of this residue in controlling the ion permeation. In K540Q mutant, even if N(CN)_2_^−^ was significantly less permeable than in wt channel, the AMFE was still present.

AMFE is generally explained as the indication that multiple ions are moving through the channel pore along a single line [44]. However, an alternative mechanism has been proposed to explain the AMFE. In this picture the AMFE depends on a local depletion of the ion concentration close to an ion binding site that reduces the current of that ion species through the pore. This model was originally proposed by Nonner et al. [45] and it can predict AMFE even when (on average) less than one ion is found in the channel pore. The AMFE in the L-type Ca^2+^ channel [46] and in the ryanodine receptor [47] could be predicted by this model. However, more experiments are still needed to fully clarify which mechanism is responsible for AMFE in TMEM16A and B.

### Blockage/activation by permeant anions

Our previous studies showed that the pore occupancy by permeant anions controlled the gating of TMEM16B [31]. Similar results were obtained in TMEM16A [43] and in native CaCCs from *Xenopus* oocytes [29] or from acinar cells of rat parotid gland [48]. Here, we report that mutation of R573 and K540 alters the activation by intracellular N(CN)_2_^−^ and reverts the blockage of C(CN)_3_^−^ with an increase of the current amplitude. These data suggest that the effect of these anions on channel conductance depend, at least partially, on the same residues controlling the permeability, confirming the strong connection between permeation and gating in the TMEM16B channel. Moreover, since this process is voltage-dependent, it indicates that both R573 and K540Q are subject to the membrane voltage field, even if further experiments will be necessary to measure precisely their position along the electric field.

### Conclusions

In conclusion, I found some evidence that R573 and K540 residues control the ion permeability of TMEM16B channel. This effect is asymmetrical across the plasma membrane and depends on the level of channel activation. Moreover, these residues contribute also to control the blockage or the activation by permeant anions. These data confirm that the proposed pore observed in the crystal structure of nhTMEM16 is involved in controlling ionic flux through the membrane. Further studies will clarify the relative contributions of the different portions of the pore in controlling ion selectivity and gating in TMEM16 ion channels.
